# Abnormal network homogeneity of default-mode network and its relationships with clinical symptoms in antipsychotic-naïve first-diagnosis schizophrenia

**DOI:** 10.3389/fnins.2022.921547

**Published:** 2022-07-25

**Authors:** Mingjun Kong, Tian Chen, Shuzhan Gao, Sulin Ni, Yidan Ming, Xintong Chai, Chenxi Ling, Xijia Xu

**Affiliations:** ^1^Department of Psychiatry, the Affiliated Brain Hospital of Nanjing Medical University, Nanjing Brain Hospital, Nanjing, China; ^2^Department of Psychiatry, Nanjing Brain Hospital, Medical School, Nanjing University, Nanjing, China

**Keywords:** schizophrenia, cognitive dysfunction, default-mode network, resting-state functional magnetic resonance imaging, network homogeneity

## Abstract

Schizophrenia is a severe mental disorder affecting around 0.5–1% of the global population. A few studies have shown the functional disconnection in the default-mode network (DMN) of schizophrenia patients. However, the findings remain discrepant. In the current study, we compared the intrinsic network organization of DMN of 57 first-diagnosis drug-naïve schizophrenia patients with 50 healthy controls (HCs) using a homogeneity network (NH) and explored the relationships of DMN with clinical characteristics of schizophrenia patients. Receiver operating characteristic (ROC) curves analysis and support vector machine (SVM) analysis were applied to calculate the accuracy of distinguishing schizophrenia patients from HCs. Our results showed that the NH values of patients were significantly higher in the left superior medial frontal gyrus (SMFG) and right cerebellum Crus I/Crus II and significantly lower in the right inferior temporal gyrus (ITG) and bilateral posterior cingulate cortex (PCC) compared to those of HCs. Additionally, negative correlations were shown between aberrant NH values in the right cerebellum Crus I/Crus II and general psychopathology scores, between NH values in the left SMFG and negative symptom scores, and between the NH values in the right ITG and speed of processing. Also, patients’ age and the NH values in the right cerebellum Crus I/Crus II and the right ITG were the predictors of performance in the social cognition test. ROC curves analysis and SVM analysis showed that a combination of NH values in the left SMFG, right ITG, and right cerebellum Crus I/Crus II could distinguish schizophrenia patients from HCs with high accuracy. The results emphasized the vital role of DMN in the neuropathological mechanisms underlying schizophrenia.

## Introduction

Schizophrenia is a chronic psychiatric syndrome impacting around 0.5–1% of the world’s population (Smigielski et al., 2020), and disturbances in sensory perception, emotion processing, thought, and social function as well as cognitive deficits, are hallmarks of schizophrenia (Jauhar et al., 2022). With complex heterogeneity in clinical manifestations and the low cure and high recurrence rates, considerable medical resources are devoted to the treatment and rehabilitation of schizophrenia patients, causing an increased economic burden on society and patients’ families. Therefore, it is important to further elucidate the potential pathological mechanisms of this disorder to develop effective therapeutic interventions.

Implicated by the disconnection hypothesis, one of the main pathological characteristics of schizophrenia is the disruption of neural synchronization and information integration. Evidence shows that network disruptions might be a biomarker of schizophrenia (van den Heuvel and Fornito, 2014). Among the empirically studied resting-state networks of schizophrenia, it is noteworthy that a set of functionally connected brain regions, comprising the medial prefrontal cortex (MPFC), bilateral posterior cingulate cortex/precuneus (PCC/PCu), lateral posterior cortices, the cerebellum Crus I and Crus II, and parts of the parietal and temporal lobe cortex and the hippocampus, of the default-mode network (DMN), play a key role in the development of schizophrenia (Guo et al., 2014a,c). Several regions of DMN are active at rest and inhibited when the brain is working (Raichle et al., 2001). Previous studies demonstrated that hyperactivity within the DMN played a role in cognitive dysfunction and psychotic symptoms of patients with schizophrenia (Buckner et al., 2008) and that the changes in the DMN were associated with diagnosis (de Filippis et al., 2019) and treatment response of antipsychotic drugs (Mehta et al., 2021; Yang et al., 2021). Healthy individuals at high risk of schizophrenia also showed abnormal levels of functional connectivity (FC) within this network (Shim et al., 2010; Dodell-Feder et al., 2014; Anteraper et al., 2020), which highlighted the importance of the DMN as a potential biomarker of the development of schizophrenia and implied that genetic factors may play a role in the pathogenesis of diseases by interacting with the strength of FC in this network.

Although neuroimaging studies have shown dysfunctions in the network of schizophrenia, the findings are inconsistent. The FC of some regions of the DMN decreased (Camchong et al., 2011) and increased in some other regions (Jamea et al., 2021). Moreover, the relationships between specific regions of the DMN and clinical symptoms also are different (Camchong et al., 2011; Jamea et al., 2021; Roig-Herrero et al., 2022). The heterogeneity of the results may be related to the heterogeneity of factors such as patient characteristics and analytical methods, including those used to analyze the DMN and assess clinical symptoms. Previous studies have widely used the seed-based region of interest (ROI) and independent component analysis (ICA) methodologies to analyze the DMN. While the ROI analysis may be biased to the selection of the predetermined seeds, ICA may fail to identify a direct relationship between extracted components and the previously defined hypothesis. Here, network homogeneity (NH), a voxel-wise measure, provides an unbiased survey of a particular network and identifies abnormal brain regions in network coherence (Uddin et al., 2008). This approach is extensively used to explore the significance of networks in the pathogenesis of psychoses (Guo et al., 2014b; Wei et al., 2016; Zhang et al., 2020), and the findings proved that NH has great potential to explore the pathological mechanisms underlying diseases, including schizophrenia.

In the current study, the FC analysis of the DMN was performed using the NH method in first-diagnosis, drug-naïve schizophrenia patients. We hypothesized that altered NH values within the DMN would be identified in patients in contrast to healthy subjects. Multiple stepwise regression analysis was performed to identify how distinctly altered NH values in these brain regions correlated to cognition dimensions and clinical symptoms differently, to provide a reference for developing better therapeutic interventions targeting specific brain regions to alleviate symptom severity of schizophrenia patients. Additionally, we applied the receiver operating characteristic (ROC) curves analysis and a machine learning approach [support vector machine (SVM)] to identify the brain regions that will help differentiate patients from the healthy subjects.

## Materials and methods

### Subjects

A total of 57 subjects with schizophrenia treated at the Affiliated Brain Hospital of Nanjing Medical University were recruited between April 2018 and December 2019. The inclusion criteria were as follows: (1) patients who were clinically diagnosed with schizophrenia by two chief psychiatrists based on the diagnostic criteria of schizophrenia of the International Classification of Diseases, 10th Revision; (2) first-diagnosis antipsychotic drug-naïve patients; (3) patients who were 16–60 years old, Han nationals, and right-handed; (4) who had a Wechsler intelligence score ≥ 70; and (5) a Positive and Negative Syndrome Scale (PANSS) (Kay et al., 1987) total scores ≥ 60. The exclusion criteria were as follows: (1) patients who had other mental disorders or any severe physical diseases or substance abuse ever; (2) severe organic brain disease or brain trauma; (3) contraindications or non-cooperation during magnetic resonance imaging (MRI).

Age- and sex-matched healthy controls (HCs) were recruited via an advertisement during the same period. The inclusion criteria were as follows: (1) no history of psychotic symptoms assessed using the MINI-International Neuropsychiatric Interview; (2) no familial history of psychiatric illness in two lines and three generations. The exclusion criteria were similar to that of the patient group.

The psychopathology and cognitive performance were measured using the PANSS for patients and MATRICS Consensus Cognitive Battery (Nuechterlein et al., 2008) for all participants. All participants signed written informed consent. The current study was approved by the local Medical Ethics Committee of the Affiliated Brain Hospital of Nanjing Medical University (2017-KY017).

### Magnetic resonance imaging acquisition

Images were obtained using a 3T Siemens MRI scanner. Participants were informed to close their eyes, stay awake, and remain motionless. The MRI scanning parameters were as follows: slice thickness = 4 mm; repetition time = 2,000 ms; field of view = 220 × 220 mm; gap = 0.6 mm; flip angle = 90°; matrix size = 64 × 64; time point = 240; echo time = 30 ms; and layers = 33.

### Data preprocessing

Data Processing Assistant for Resting-State Functional MRI (DPARSF) in MATLAB (Mathworks) was applied to preprocess the MRI data. If the maximal translation of the participants was over 3 mm and maximal rotation was over 3° in x, y, or z axes after slice timing and head motion correction, the images were excluded. Next, the motion-corrected functional volumes were spatially normalized to the Montreal Neurological Institute (MNI) space and resampled to 3 mm × 3 mm × 3 mm. After normalization, the transformed images were temporally bandpass filtered (0.01–0.08 Hz) and were linearly detrended. Several spurious covariates, including the signal from the 24 head motion parameters acquired by rigid body correction, ventricular seed-based ROI, and the white matter-centered brain region, were removed. The global signal was preserved for further analyses (Hahamy et al., 2014).

### Default-mode network identification

The group ICA, in the GIFT toolbox^1^, was performed for all subjects (Liu C. H. et al., 2012; Guo et al., 2013). The three main steps followed in the analysis were as follows: (1) reduction of data; (2) the minimum description length criterion was set to 20 to estimate separation of independent components; (3) back reconstruction. Finally, the generated DMN mask was applied in the following NH analyses (Raichle, 2015). More details are provided in the Supplementary Methods [Default-Mode Network (DMN) identification].

### Network homogeneity analysis

We carried out NH analysis using MATLAB software (Mathworks). For a given voxel with others in a particular whole-brain network, the time series similarity is defined as homogeneity, and the NH value of a voxel is its mean correlation coefficient. The average correlation coefficients were transformed in *z*-values using Fisher *r*-to-*z* transformation (Buckner et al., 2009) to generate the NH maps after being smoothened using a Gaussian kernel of 8-mm full-width at half-maximum for further analyses. Age, sex, and education were regarded as confounders. The two-sample *t*-test via voxel-wise cross-subject statistics was applied to calculate the differences in NH in the DMN between patients and HCs. A corrected *p*-value < 0.05 indicated significance for multiple comparisons using the Gaussian Random Field approach (voxel significance, *p* < 0.001; cluster significance, *p* < 0.05).

### Statistical analyses

For the demographic and clinical data, the continuous variables were compared using a two-sample independent *t*-test between patients and HCs. A Chi-square test was employed to identify gender differences. Significance was indicated by a two-tailed *p*-value < 0.05.

Region of interest were brain regions with aberrant NH values. Mean NH values in these ROIs were calculated for stepwise multiple regression analysis between the NH values in abnormal brain regions and the PANSS scores as well as the cognitive dimension scores, with the aberrant NH values in the DMN regions, age, education, and illness duration as independent variables, and the scores of the subdimensions of PANSS as well as all subsets of cognitive performance as dependent variables in the patient group. The statistical analyses were performed using the Statistical Package for Social Science version 25.0 (SPSS 25.0).

### Classification analysis using receiver operating characteristic and support vector machine

Receiver operating characteristic analyses were conducted using SPSS 25.0. The values of Sensitivity + Specificity – 1 were defined as the Youden index to identify the cut-off points. Patients could be correctly distinguished from the HCs with optimal sensitivity and specificity using the cut-off points.

To further improve the accuracy of classification, we employed SVM, a method of supervised learning, to test the feasibility and effectiveness of abnormal NH values in the brain regions to differentiate patients from HCs using the LIBSVM software package^2^. The LIBSVM software used the leave-one-out method. The grid search method was applied to search the optimal parameters of the classification model with aberrant NH values in the DMN regions to discriminating patients from HCs. More details are provided in the Supplementary Methods [Classification analysis using support vector machine (SVM)].

## Results

### Demographic distribution and clinical information of subjects

There was no difference between the patients with schizophrenia and HCs in age (*t*-test, *t* = 1.808, df = 105, *p* = 0.074) and gender distribution (Chi-square test, χ2 = 1.320, df = 1, *p* = 0.251). However, the years of education of HCs were higher than those of patients (*t*-test, *t* = –5.018, df = 105, *p* = 0.000).

No difference was found in social cognition as well as working memory between patients with schizophrenia and HCs. However, other parameters of cognitive performance were poor in patients with schizophrenia compared to HCs (Table 1).

**TABLE 1:** Characteristics of the subjects.

	Patients (*n* = 57)	HCs (*n* = 50)	t/x^2^	*P*
Age (years)	31.63 ± 11.43	28.38 ± 6.87	1.81	0.074^b^
Education (years)	12.86 ± 3.42	15.64 ± 2.26	–5.02	0.000^b^
Gender (male/female)	20/37	23/27	1.32	0.251^a^
Speed of processing	34.70 ± 12.12	44.28 ± 9.10	–4.57	0.000^b^
Attention/Vigilance	38.19 ± 13.61	45.56 ± 9.69	–3.25	0.002^b^
Verbal Learning	37.67 ± 13.66	44.52 ± 7.51	–3.27	0.002^b^
Visual Learning	42.05 ± 11.61	47.16 ± 9.50	–2.50	0.014^b^
Reasoning and Problem Solving	38.84 ± 10.78	45.44 ± 9.91	–3.28	0.001^b^
Working Memory	32.51 ± 12.32	34.28 ± 11.22	–0.77	0.441^b^
Social Cognition	33.14 ± 8.15	35.32 ± 6.49	–1.52	0.132^b^
Overall Composite	28.47 ± 13.45	37.60 ± 9.19	–4.14	0.000^b^
Duration (years)	2.41 ± 2.70			
PANSS				
Positive symptoms	26.39 ± 4.85			
Negative symptoms	20.68 ± 6.89			
General psychopathology	44.79 ± 7.41			
Total	91.84 ± 14.16			

HCs, healthy controls; PANSS, Positive and Negative Syndrome Scale.

^a^The p-value was obtained by x^2^ test.

^b^The p-value was gained by two-sample independent t-tests.

### Default-mode network mask

**FIGURE 1:**
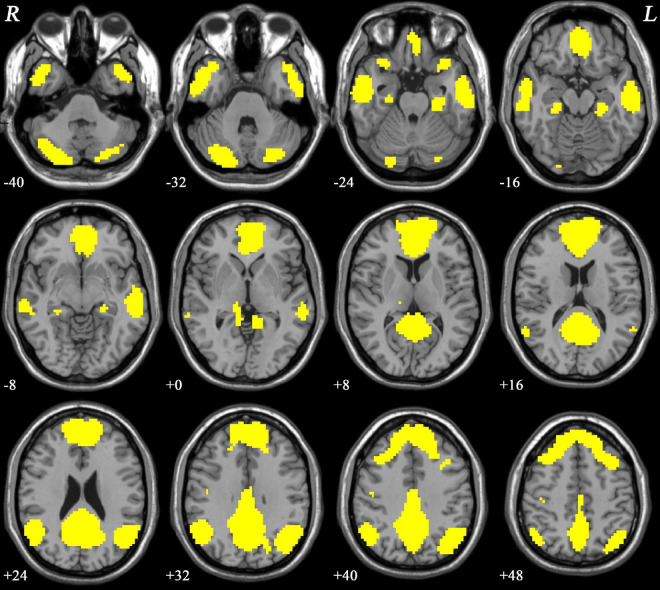
The DMN mask was determined using ICA. R and L denote the right and left sides, respectively; DMN, default-mode network; ICA, independent component analysis.

The DMN mask was identified with a template mask using the group ICA method for all participants. The DMN consisted of the ventral anterior cingulate cortex (ACC), bilateral MPFC, PCC/PCu, lateral temporal cortex, cerebellum Crus I/Crus II, and lateral, medial, and inferior parietal lobes (Figure 1). The obtained DMN mask was used for NH analyses.

### Differences in network homogeneity between patients with schizophrenia and healthy controls

**FIGURE 2:**
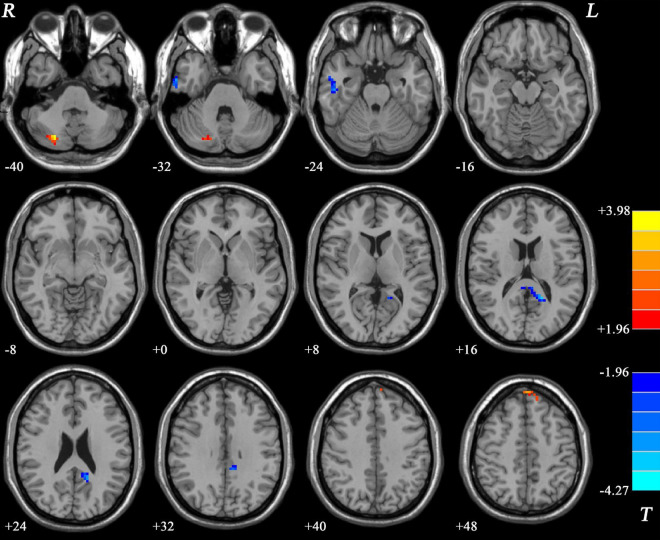
Differences in the NH values in the brain regions between patients and HCs. The color of the bars denotes the *t*-values (two-sample *t*-tests). Blue and red represent lower and higher NH, respectively. R and L denote the right and left sides, respectively; HCs, healthy controls; NH, network homogeneity.

As shown in Figure 2 and Table 2, significant differences in NH values on the DMN mask were identified using the voxel-wise cross-subject comparisons. Compared to HCs, schizophrenia patients showed higher NH values in the left superior medial frontal gyrus (SMFG) and right cerebellum Crus I/Crus II and lower NH values in the right inferior temporal gyrus (ITG) and bilateral PCC.

**TABLE 2:** Differences in DMN NH values between groups.

Cluster location	Peak (MNI)			Number of voxels	*T* value
	*x*	*y*	*z*		
Left SMFG	–3	54	45	30	3.5043
Right Cerebellum Crus I and II	24	–75	–39	45	3.9834
Bilateral PCC	–18	–51	15	90	–3.6884
Right ITG	51	–18	–27	63	–3.7776

SMFG, superior medial frontal gyrus; PCC, posterior cingulate cortex; ITG, inferior temporal gyrus; MNI, Montreal Neurological Institute; NH, network homogeneity; DMN, default-mode network.

### Correlations between clinical characteristics and network homogeneity values in the brain regions in patients

**FIGURE 3:**
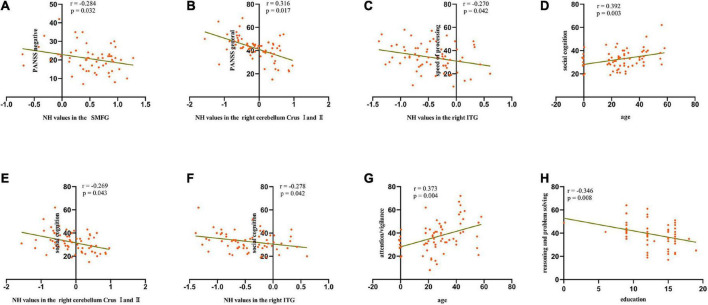
Scatterplots of significant associations between NH values in the SMFG and negative symptom scores **(A)**, NH values in the right cerebellum Crus I/Crus II and general psychopathology scores **(B)**, NH values in the right ITG and speed of processing scores **(C)**, age and social cognition scores **(D)**, NH values in the right cerebellum Crus I/Crus II and social cognition scores **(E)**, NH values in the right ITG and social cognition scores **(F)**, age and attention/vigilance scores **(G)**, and education and reasoning and problem-solving scores **(H)** in the patient group. SMFG, superior medial frontal gyrus; ITG, inferior temporal gyrus; PANSS, Positive and Negative Syndrome Scale; NH, network homogeneity.

We calculated the average NH values in the left SMFG, right cerebellum Crus I/Crus II, bilateral PCC, and right ITG. As shown in Figure 3 and Table 3, significant negative correlations were observed between aberrant NH values in the right cerebellum Crus I/Crus II and general psychopathology scores (standardized β coefficients = –0.316, *p* = 0017), between NH values in the left SMFG and negative symptom scores (standardized β coefficients = –0.284, *p* = 0.032), and between NH values in the right ITG and speed of processing (standardized β coefficients = –0.270, *p* = 0.042). Moreover, the patients’ age and the NH values in the right cerebellum Crus I/Crus II and the right ITG were the predictors of performance in social cognition test (standardized β coefficients = 0.368, *p* = 0.002; standardized β coefficients = –0.319, *p* = 0.008; standardized β coefficients = –0.286, *p* = 0.017, respectively). Additionally, age explained 12.3% of the variance in attention/vigilance (standardized β coefficients = 0.373, *p* = 0.004), and education accounted for 10.4% of the variance in reasoning and problem solving (standardized β coefficients = –0.346, *p* = 0.008).

**TABLE 3:** Multiple stepwise regression analysis between the abnormal NH values in the brain regions and PANSS dimensions and cognitive tests.

Dependent variable					Predictive variables		
	Adj *R*^2^	*B*	*F*	*P*	Variable	Standardized β	*P*
PANSS							
Negative symptoms	0.081	22.89	4.83	0.032	Left SMFG	–0.284	0.032
General psychopathology	0.083	43.76	6.10	0.017	Right Cerebellum Crus I and II	–0.316	0.017
Speed of processing	0.056	31.07	4.33	0.042	Right ITG	–0.270	0.042
Attention/Vigilance	0.123	24.15	8.89	0.004	Age	0.373	0.004
Reasoning and Problem Solving	0.104	52.87	7.48	0.008	Education	–0.346	0.008
Social Cognition	0.270	21.11	7.92	0.000	Age	0.368	0.002
					Right Cerebellum Crus I and II	–0.319	0.008
					Right ITG	–0.286	0.017

SMFG, superior medial frontal gyrus; ITG, inferior temporal gyrus; PANSS, Positive and Negative Syndrome Scale; NH, network homogeneity.

### Classification results to differentiate patients from healthy controls

**FIGURE 4:**
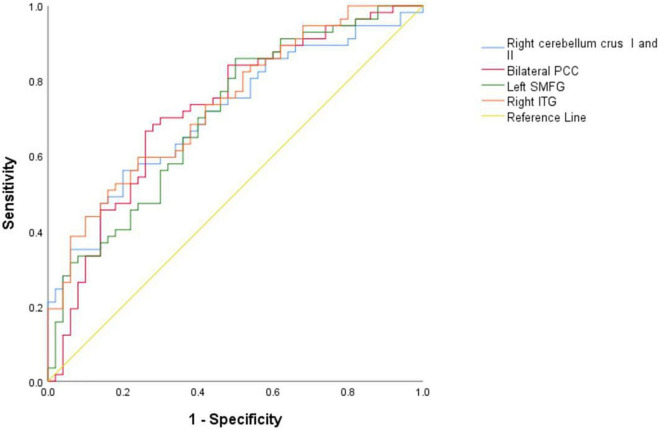
Results of the ROC analyses used to differentiate between patients and HCs using the NH values in the different brain regions. NH, network homogeneity; HCs, healthy controls; PCC, posterior cingulate cortex; SMFG, superior medial frontal gyrus; ITG, inferior temporal gyrus; ROC, receiver operating characteristic.

As shown in Table 4 and Figure 4, the brain areas with abnormal NH values could distinguish patients from HCs with a relatively high degree of accuracy using ROC analysis. Our results indicated that the NH values in bilateral PCC with an accuracy of 72.30%, a sensitivity of 66.70%, and a specificity of 74.00% discriminated schizophrenia patients from healthy subjects. Moreover, the optimal accuracy, specificity, and sensitivity of the NH values in the left SMFG were 70.70, 50.00, and 86.00%, respectively and those in the right ITG were 73.80, 76.00, and 59.60%, respectively. The right cerebellum Crus I/Crus II NH values showed an accuracy of 71.20%, a specificity of 80.00%, and a sensitivity of 56.10% in differentiating schizophrenia patients from healthy individuals.

**TABLE 4:** ROC analyses for differentiating the schizophrenia patients from the HCs.

Brain regions	Area under the curve	Cut-off point	Sensitivity	Specificity
Right cerebellum Crus I and II	0.712	0.5852	56.10%	80.00%
Bilateral PCC	0.723	0.5622	66.70%	74.00%
Left SMFG	0.707	0.4571	86.00%	50.00%
Right ITG	0.738	0.5678	59.60%	76.00%

SMFG, superior medial frontal gyrus; ITG, inferior temporal gyrus; PCC, posterior cingulate cortex; HCs, healthy controls; ROC, receiver operating characteristic.

**FIGURE 5:**
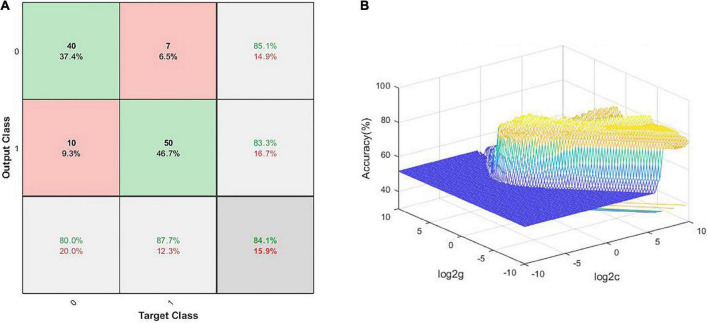
Visualization of classification using SVM analysis with the NH values in the combined brain regions, comprising the left SMFG, right cerebellum Crus I/Crus II, and right ITG; **(A)** confusion matrix; **(B)** SVM parameter results of 3D view. Target Class and Output Class represent actual and predicted results of classification, respectively; SMFG, superior medial frontal gyrus; ITG, inferior temporal gyrus; NH, network homogeneity; SVM, support vector machine.

The SVM results demonstrated that the NH values in the combined brain regions of the right cerebellum Crus I/Crus II, right ITG, and left SMFG showed an optimal accuracy of 84.11% to distinguish patients from HCs (Table 5 and Figure 5).

**TABLE 5:** The results of SVM to classify patients from HCs.

Feature	Accuracy (%)	Feature	Accuracy (%)
1	65.42	123	79.44
2	69.16	124	74.77
3	65.42	134	84.11
4	64.49	234	76.64
12	72.90	1234	74.77
13	72.90		
14	70.09		
23	74.77		
24	73.83		
34	77.57		

1, 2, 3, 4 represent right cerebellum Crus I and II, bilateral posterior cingulate cortex, left superior medial frontal gyrus, right inferior temporal gyrus, respectively. SVM, support vector machine.

## Discussion

Our present study demonstrated that the patients with schizophrenia exhibited increased NH values in the left SMFG and right cerebellum Crus I/Crus II, and reduced NH values in the bilateral PCC and right ITG compared to HCs. Moreover, for patients with schizophrenia, we observed negative correlations between the NH values in the left SMFG and negative symptom scores, between the NH values in the right cerebellum Crus I/Crus II and general psychopathology scores, and between the right ITG and the speed of processing scores. Age, NH values in the right cerebellum Crus I/Crus II and the right ITG significantly contributed to the social cognition performance in schizophrenia patients. In conclusion, the correlations between the NH values in these regions and the symptom/cognition dimensions implied that the poor-level coordination in the DMN may be responsible for deficits in cognitive performance and symptoms to some extent.

As a core node of DMN, the MPFC abnormalities have been considered an intrinsic feature of schizophrenia and found to be related to severe psychiatric symptoms like deficits in cognition, especially the execution control function (Li et al., 2019). Our study showed increased NH values in the left SMFG. Zhang et al. (2020) also found increased NH values in the left MPFC, which may help distinguish schizophrenic patients from HCs. In contrast to our results, previous studies also found reduced NH values in left MPFC in the DMN (Guo et al., 2014c) or no difference in NH values in MPFC at baseline in patients with schizophrenia compared to HCs, but after 6 months of treatment with Olanzapine, NH in the left superior MPFC increased in the patient group (Guo et al., 2017). The difference in results may be associated with the heterogeneity of patients with schizophrenia, such as age and sex, illness duration, clinical characteristics, and so on. Moreover, our study showed a negative correlation between the NH values in the left SMFG and the scores of negative symptoms. Also, longitudinal brain analysis to elucidate the effects of drug treatment showed that antipsychotic drugs can regulate the functional and connectional integrity of this region to improve the severity of psychotic symptoms, and the levels of FC of the bilateral superior MPFC at baseline could predict the effectiveness of treatments (Guo et al., 2017; Shan et al., 2020). Studies using magnetic resonance spectroscopy further showed that the improvement in symptoms was accompanied by changes in the levels of γ-aminobutyric acid neurotransmitters in the MPFC brain region after antipsychotic drug treatment (Li et al., 2022), further verifying that functional disconnection in MPFC within the whole DMN was involved in the manifestation of clinical symptoms. Taken together, further therapeutic measures targeting the specific brain region containing MPFC are important to improve psychotic symptoms of schizophrenia.

Consistent with the previous reports (Lee et al., 2016), our findings showed a negative correlation between the abnormal neural activity of ITG and neurocognitive performance, especially in speed of processing and social cognition. Using the NH method, previous studies showed no difference between patients with schizophrenia and HCs in the right ITG (Guo et al., 2014c,^2017^; Shan et al., 2020; Zhang et al., 2020); however, we found that the NH values in right ITG reduced in the patients. The difference in results may be associated with the heterogeneity of patients with schizophrenia. Nevertheless, research comprising functional or structural MRI using different analyses, such as global-brain FC (Zhao et al., 2022), the dynamic amplitude of low-frequency fluctuation (Wang et al., 2021), full- and short-range strength of FC (Miao et al., 2020), and Trace (Lee et al., 2016), have observed aberrant ITG in patients with schizophrenia, which is associated with psychotic (Lee et al., 2016) and cognitive symptoms (Lee et al., 2016) and might predict the response to an antipsychotic drug after 8 weeks (Zhu et al., 2018). Previous studies also showed that both patients with schizophrenia and their unaffected siblings shared similar alterations in the ITG (Liu H. et al., 2012; Zhu et al., 2018), and the neural activity of ITG was regulated by regulating by the Disrupted-in-Schizophrenia-1 gene (Gou et al., 2018), suggesting that the ITG might be a potential biomarker of endophenotype for schizophrenia. Zhu et al. (2020) stated that the right ITG might show unique abnormalities in patients with schizophrenia compared with those with bipolar disorder and attention-deficit/hyperactivity disorder. Moreover, Miao et al. (2020) showed that the functional impairment in this region might be an ongoing pathological process in schizophrenia patients, and it is barely affected by antipsychotic drugs. Above all, to a certain extent, our results provided diverse findings regarding ITG and novel insights into exploring symptomatic and cognitive-related mechanisms in patients with schizophrenia.

In addition to being engaged in motor control and coordination, the cerebellum also plays an important role in emotion and cognitive processing (Stoodley et al., 2012; Sokolov et al., 2017). In line with the results in our study, higher NH values in the right cerebellum Crus I (Guo et al., 2014c) and right cerebellum Crus II (Shan et al., 2020) have been reported in schizophrenia patients. Further, our results of stepwise regression analysis showed that abnormal NH values in both these brain regions were associated with general psychopathology and social cognition. Kuhn et al. (2012) showed that gray matter volume of left cerebellum Crus I/Crus II was related to thought disorder and Trail-making test B. The reduction in general psychopathology was associated with the gray matter volume in the cerebellum (Crus I) (Premkumar et al., 2009), further supporting our results that cerebellum Crus I/Crus II might participate in the pathological mechanism of schizophrenia. The differences among studies might be associated with the heterogeneity of patients and the analysis methods. Laidi et al. (2019) demonstrated that patients with schizophrenia showed a decrease in the cerebellum (Crus II), whereas there was no corresponding alternation in patients with bipolar disorder, indicating that the abnormalities in cerebellum Crus I and/or Crus II might be specific to schizophrenia. According to these reports and our results of SVM, combined with SMFG, right ITG, and right cerebellum Crus I/Crus II might help distinguish between patients with schizophrenia and HCs.

Posterior cingulate cortex, as one of the important nodes of the DMN and limbic system, was associated with cognition, psychotic symptoms, and micro-RNA 137 (Zhang et al., 2018), which might participate in the pathological mechanism of schizophrenia (Leech and Sharp, 2014). Our results showed lower NH values in PCC, which is consistent with previous studies (Shan et al., 2020; Zhang et al., 2020) but in contrast to the study by Guo et al. (2014c). The difference might be related to the heterogeneity of patients with schizophrenia. Several reports showed abnormalities in PCC using different analysis methods in patients with schizophrenia, including aberrant DMN connectivity strength (Hilland et al., 2022), increased global-brain functional connectivity (Ding et al., 2019), and so on. Despite the differences in results, both the studies support that the PCC is involved in the pathological mechanism of schizophrenia. Moreover, PCC is rich in *N*-methyl-D-aspartate (NMDA) receptors (Ma and Leung, 2018), whereas phencyclidine (PCP), an NMDA receptor antagonist, is regarded as a pharmacological model of schizophrenia. He et al. (2006) showed that quetiapine might ameliorate the apoptosis in PCC induced by PCP, implicating that PCC may be a potential target for antipsychotic drugs, such as quetiapine.

### Limitations

Several limitations must be taken into account for this study. Firstly, owing to the small sample size, the results herein, cannot be extrapolated to the general population. Secondly, the DMN mask extracted from all the participants using ICA may have affected the analyses (Uddin et al., 2008). Thirdly, we may have neglected the pathophysiology in the other brain regions or networks by focusing on the connective dysfunction of the DMN. Finally, the years of education of schizophrenia patients were different from those of HCs, which may have an effect on the cognitive performance, especially reasoning and problem solving, in schizophrenia patients. The age of patients also affected the clinical characteristics in the patient group although there were no differences in age between patients and HCs. Future studies should consider these confounding effects.

## Conclusion

Despite its limitations, our study revealed significantly aberrant intrinsic network organization of the DMN in schizophrenia and demonstrated that the combination of NH values in the SMFG, right ITG, and right cerebellum Crus I/Crus II might help distinguish between patients with schizophrenia and HCs and can be regarded as an underlying biomarker.

## Data availability statement

The original contributions presented in this study are included in the article/Supplementary Material, further inquiries can be directed to the corresponding author.

## Ethics statement

The studies involving human participants were reviewed and approved by Medical Ethics Committee of The Affiliated Nanjing Brain Hospital, Nanjing Medical University (2017-KY017). Written informed consent to participate in this study was provided by the participants or their legal guardian/next of kin. Written informed consent was obtained from the individual(s), and minor(s)’ legal guardian/next of kin, for the publication of any potentially identifiable images or data included in this article.

## Author contributions

MK wrote the first draft of this manuscript. XX designed the research. SG, TC, MK, YM, SN, XC, and CL collected the clinical and imaging data. XX, MK, and TC analyzed the data. All the authors reviewed and approved the final version of the manuscript.

## Footnotes

1
http://mialab.mrn.org/software/

2
http://www.csie.ntu.edu.tw/c̃jlin/libsvm/
