# Supplementary material for: Abnormal network homogeneity of default-mode network and its relationships with clinical symptoms in antipsychotic-naïve first-diagnosis schizophrenia
Source: Front Neurosci. 2022 Jul 25;16:921547. doi: 10.3389/fnins.2022.921547 (PMC9369006; doi:10.3389/fnins.2022.921547)
Supplement: Supplementary file 1 [file Presentation_1.zip › Supplementary Material Presentation/Supplementary Methods.docx]

**Supplementary Methods**

**Default-Mode Network (DMN) identification**

First, we acquired the lower dimensions using subject- and group-level principal component analyses (PCA) and evaluated the number of independent components (ICs) by the minimum description length criterion, set to 20 in this study(Guo et al., 2013;Liu et al., 2012). Second, specific ICs were obtained using a back-reconstruction strategy based on the group ICs and PCA reduction results(Erhardt et al., 2011). According to the templates by the GIFT, two DMN components were chosen. Finally, statistical mapping was performed using voxel-wise one-sample t-tests for each component (the corrected p < 0.05 for multiple comparisons using the Gaussian Random Field approach, voxel significance: p < 0.001, cluster significance: p < 0.05)(Song et al., 2011). The two masks were combined to generate a DMN mask used in further analyses(Raichle 2015).

**Classification analysis using support vector machine (SVM)**

The basic idea of SVM is to find the optimal line and optimal surface with the largest interval through appropriate kernel function, and classify the data. In this study, SVM was performed to distinguish schizophrenia patients from healthy controls using the network homogeneity values in the abnormal DMN regions, using the LIBSVM package (http://www.csie.ntu.edu.tw/~cjlin/libsvm/). The LIBSVM software applied the leave-one-out (LOO) method and optimal sensitivity and specificity were obtained through cross-validation. The kernel type was a Gaussian kernel. First, sensitivity and specificity were used to separate all the individuals with class labels and establish the decision function. Decision functions are conducted to predict the results of a classification in new samples. According to the LOO verification, given a data set containing X (number of cases) samples, X iterations were performed. In each iteration, we calculated the classifier through the X-1 samples, and the remaining samples were tested.

**References**

Erhardt, E. B., Rachakonda S., Bedrick E. J., Allen E. A., Adali T., Calhoun V. D. (2011). Comparison of multi-subject ICA methods for analysis of fMRI data. Hum Brain Mapp. 32**,** 2075-2095. doi: 10.1002/hbm.21170.

Guo, W., Liu F., Zhang J., Zhang Z., Yu L., Liu J., et al. (2013). Dissociation of regional activity in the default mode network in first-episode, drug-naive major depressive disorder at rest. J Affect Disord. 151**,** 1097-1101. doi: 10.1016/j.jad.2013.09.003.

Liu, C. H., Ma X., Li F., Wang Y. J., Tie C. L., Li S. F., et al. (2012). Regional homogeneity within the default mode network in bipolar depression: a resting-state functional magnetic resonance imaging study. PLoS One. 7**,** e48181. doi: 10.1371/journal.pone.0048181.

Raichle, M. E. (2015). The brain's default mode network. Annu Rev Neurosci. 38**,** 433-447. doi: 10.1146/annurev-neuro-071013-014030.

Song, X. W., Dong Z. Y., Long X. Y., Li S. F., Zuo X. N., Zhu C. Z., et al. (2011). REST: a toolkit for resting-state functional magnetic resonance imaging data processing. PLoS One. 6**,** e25031. doi: 10.1371/journal.pone.0025031.
